# Removal of trivalent chromium from aqueous solution using aluminum oxide hydroxide

**DOI:** 10.1186/s40064-016-2983-x

**Published:** 2016-08-08

**Authors:** Agaje Bedemo, Bhagwan Singh Chandravanshi, Feleke Zewge

**Affiliations:** Department of Chemistry, College of Natural Sciences, Addis Ababa University, P.O. Box 1176, Addis Ababa, Ethiopia

**Keywords:** Aluminum oxide hydroxide, Adsorption of chromium, Chromium removal, Tannery wastewater

## Abstract

Water is second most essential for human being. Contamination of water makes it unsuitable for human consumption. Chromium ion is released to water bodies from various industries having high toxicity which affects the biota life in these waters. In this study aluminum oxide hydroxide was tested for its efficiency to remove trivalent chromium from aqueous solutions through batch mode experiments. Chromium concentrations in aqueous solutions and tannery waste water before and after adsorption experiments were determined using flame atomic absorption spectrometry. The effects of pH, contact time, initial concentration and adsorbent dosage on the adsorption of Cr(III) were studied. The study revealed that more than 99 % removal of Cr(III) was achieved over wide range of initial pH (3–10). The optimum conditions for the removal of Cr(III) were found to be at pH 4–6 with 40 g/L adsorbent dose at 60 min of contact time. The adsorption capacity was assessed using Langmuir and Freundlich isotherms. The equilibrium data at varying adsorbent dose obeyed the two isotherms. The adsorbent was found to be efficient for the removal of Cr(III) from tannery waste effluent.

## Background

Heavy metals are toxic even at trace levels and their presence in the environment is of major concern to many forms of life on the earth (Selvaraj et al. 2003; Igwe and Abia 2006). Most metal contamination comes to the aqueous environments through various industrial processes. Toxic metals are not biodegradable and hence their concentrations need to be reduced to acceptable levels prior to discharge of industrial effluents to the water bodies. World Health Organization (WHO) has considered aluminum, chromium, manganese, iron, cobalt, nickel, copper, zinc, cadmium, mercury and lead to be of most immediate concern (Baig et al. 2003).

Chromium compounds are widely used in many industries, resulting in disposal of large amounts chromium ions into the environment. Chromium contamination of soil and water is a major environmental problem. The toxicity of chromium varies greatly among a wide variety of chromium compounds, its oxidation state and its solubility in water (Salimi et al. 2006). Chromium occurs in the environment primarily in two valence states, trivalent chromium, Cr(III) and hexavalent chromium, Cr(VI). Chromium(III) is considered to be an essential dietary element to the human and mammals while chromium(VI) is highly toxic and possesses mutagenic and carcinogenic activity (Nomanbhay and Palanisamy 2005; Sarin and Pant 2006). However, less toxic trivalent chromium can easily be oxidized to hexavalent chromium in the presence of oxidizing impurities.

Chromium contamination may occur in the natural environment or due to industrial processes. However, it is human-caused chromium contamination that has recently been the focus of much scientific discussion, regulatory concern, and legal posturing. Chromium contamination of water bodies is the questions that continue to arise regarding the safety of the drinking water supply. Chromium from anthropogenic sources is commonly released as Cr-bearing liquid or solid wastes which may contain any combination of Cr(III) or Cr(VI) with various solubility. The concentration of Cr in discharged effluents depend mainly on the amount and state of Cr compounds utilized in the industrial process, on the pH, and on the presence of other organic and inorganic processing wastes.

Cr(III) is the primary form of Cr in water bodies and can be retained by many adsorbent. Adsorption of Cr(III) is enhanced as pH increases due to deprotonation of adsorbent surface. Adsorption of Cr(III) is usually enhanced, if the adsorbent has a high organic content as more sites are present for sorption to occur (Igwe and Abia 2006). Cr(VI) behaves like an anion. In general, sorption of Cr(VI) decreases with increasing pH while at low pH values, surfaces will be neutral or positively charged, leading to higher adsorption due opposite charge attraction. Furthermore, sorption of Cr(VI) decreases as the concentration of competing anions sorbed to solid surface increases and finally adsorption of Cr(VI) becomes negligible. Therefore, adsorption processes are used indirectly to remediate Cr(VI), i.e. Cr(VI) is first reduced to Cr(III) (Selvaraj et al. 2003).

Adsorption is an efficient and cost-effective method of chromium removal from water and wastewater. Recently the use of low-cost adsorbents has been considered to reduce chromium concentration from industrial waste effluents. Several adsorbents (eucalyptus bark, saw dust, sand, clay mineral, charcoal, and various agricultural by products like peanut shell, wheat husk, sugarcane bagasse, biosorption, magnetic beads, carbon nanotubes, *Sinorhizobium meliloti* 1021 exopolysaccharide, polyacrylic acid, ionic polyamino acid block copolymers, albumins, synthetic polyacrylamide, etc.) have been examined by many scientists for their chromium removal efficiencies in different parts of the world (Gupta et al. 2001; Garg et al. 2004; Mohan et al. 2006; da Fonseca et al. 2006; Dubey and Gopal 2007; Karnitz et al. 2007; Karale et al. 2007; Li et al. 2008; Owlad et al. 2009; Atieh et al. 2010; Ramakrishnaiah and Prathima 2012; Wiśniewska and Szewczuk-Karpisz 2013; Szewczuk-Karpisz et al. 2014; Mandal et al. 2015; Ostolska and Wiśniewska 2015; Szewczuk-Karpisz and Wiśniewska 2015; Wisniewska et al. 2016). However, there is no report in the literature on the chromium removal using local adsorbents in Ethiopia. Hence, this study focused on using locally available adsorbent with better efficiency.

## Methods

### Chemicals and reagents

All the solutions were prepared in distilled and de-ionized water. Stock standard solution of chromium metal (1000 mg/L, Buck Scientific Puro-Graphic, USA), prepared as nitrate in 2 % HNO_3_, was used as calibration standard for determination of chromium concentration using flame atomic absorption spectrometer. Stock solution of Cr(III) at a concentration of 1000 mg/L was prepared by dissolving 5.1240 g of analytically pure CrCl_3_·6H_2_O (Riedel-deHaen, Germany) in 1 L volumetric flask and diluted to the mark with distilled de-ionized water. Further working solutions were made by appropriate dilutions. 0.2 M NaOH (99.0 %, BDH Chemicals Ltd, England) and 0.2 M HCl (36 %, Fisher Scientific UK Limited, England) were used to adjust pH.

### Adsorbent

The adsorbent (aluminum oxide hydroxide) was prepared by mixing 100 g of aluminum sulfate in 500 mL of distilled water while stirring with magnetic stirrer. The resulting lower pH of 2.72 was adjusted to pH of 7.00 using 2 M NaOH. The precipitated solid material was filtered and dried at 50 °C in an oven. Then, the fractions of the dried material were treated at temperature of 200 °C using a furnace (Carbolite, ELF 11/14B Model, England). The heated adsorbents were cooled in a desiccater until its use. Aluminum sulfate (99 %) used for preparation of aluminum oxide hydroxide was obtained from the Addis Ababa Water Supply and Sewerage Authority that is produced locally by Awash Melkasa Aluminum Sulfate and Sulfuric Acid Factory (Awash Melkasa, Ethiopia).

### Instrumentation

Studying chromium removal efficiency of adsorbents involves determination of amount of chromium in the effluent solutions before and after adsorption takes place. This was done using Flame Atomic Absorption Spectrometer (Buck Scientific Model 210 VGP, East Norwalk, USA) equipped with deuterium ark background corrector, nebulizer and chromium hallow cathode lamp using air-acetylene flame.

To determine concentration of chromium in the filtrates, a series of standard chromium solutions in the range of 0.05–3 mg/L were prepared by diluting the stock solution of chromium with de-ionized water. A blank (distilled de-ionized water) and standards were run in flame atomic absorption spectrometer and four points calibration curves were established. Then, sample solutions were aspirated in to the AAS instrument and direct readings of total chromium concentrations were recorded. Three replicate determinations were carried out on each sample. The amount of Cr(III) adsorbed was then calculated from the difference between the amount before and after adsorption.

### Adsorption experiment

Batch mode experiments were carried out in 250 mL conical flask at room temperature (22 ± 2 °C). Chromium adsorption efficiency (% removal) as a function of equilibrium time, pH, amount of adsorbent and initial concentration of Cr(III) was studied. In order to optimize the contact time, 2 g of the adsorbent was stirred with 50 mL of 40 mg/L of Cr(III) solution at different time intervals (0, 2, 5, 10, 15, 20, 25, 30, 40, 50, 60, 80, and 100 min). At the end of the stirring period the samples were centrifuged at 8000 rpm for 10 min and filtered through Whatman No. 1 filter paper. The concentrations of Cr(III) in the filtrate were determined using flame atomic absorption spectrometer. To study the effect of pH, the initial pH of 50 mL of 40 mg/L Cr(III) solutions were adjusted to different pH values (2, 3, 4, 5, 6, 7, 8, 9 and 10) using 0.2 M NaOH and 0.2 M HCl. Then, 1 g adsorbent was equilibrated with these solutions for 60 min and the filtrates were analysed for Cr(III) concentration. The effect of adsorbent dosage was also studied by varying the amount of adsorbent (0.2, 0.4, 0.6, 0.8, 1.0, 1.2, 1.4, 1.8, 2.2, 2.6, 3.0, and 3 g) on an initial concentration of 40 mg/L Cr(III) at pH 3.8 for a contact time of 60 min. In another set of experiment 50 mL of Cr(III) solutions at varying concentrations (0, 10, 20, 40, 60, 80, 100 mg/L) were transferred into the conical flask containing 1 g of the adsorbent and stirred for 60 min and the filtrates were analysed for Cr(III) concentration.

## Results and discussion

All the adsorption experiments were carried out in triplicate and the data presented in all the figures are the mean values of triplicate measurements. The blank runs (before the addition of adsorbent) were also carried out in triplicate. The relative standard deviations associated with the blank runs, adsorption experiments and in the determination of Cr(III) were less 5 %.

### Effect of contact time

**Fig. 1:**
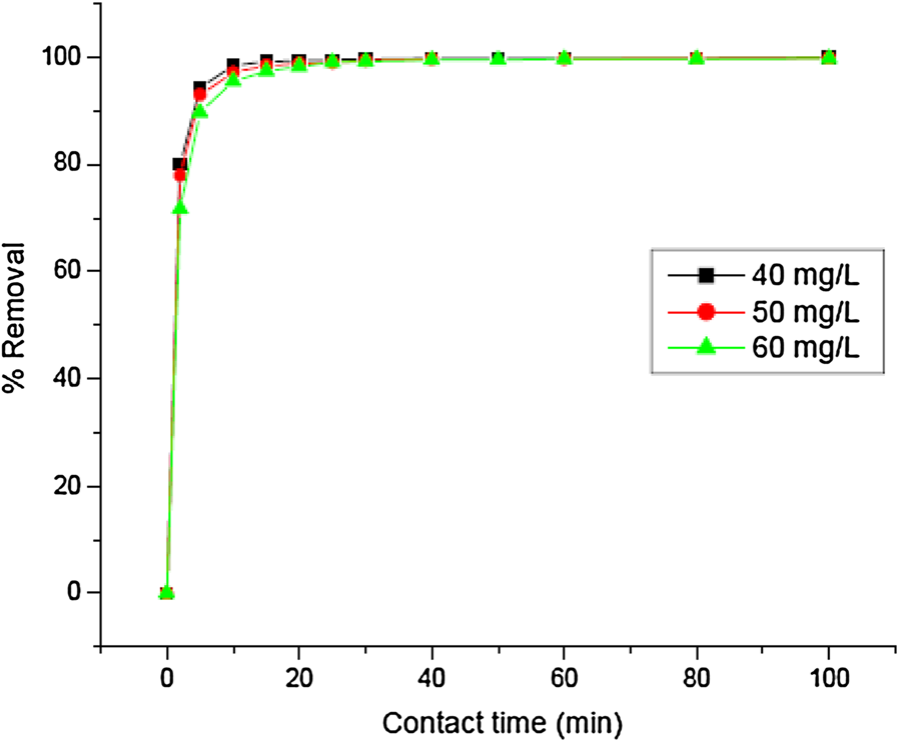
Percent removal of Cr(III) as a function of contact time (adsorbent dose 40 g/L, initial pH 3.8) at three different initial concentrations (40, 50, 60 mg/L) of Cr(III)

Adsorption experiments were carried out at an initial concentration of 40, 50 and 60 mg/L Cr(III) with 2 g adsorbent at different time intervals (2–100 min) to optimize equilibrium time for the removal of Cr(III). The plot of percent removal of Cr(III) at the three initial concentrations at different time intervals (Fig. 1) reveals that the rate of percent Cr(III) removal is higher at the beginning. This is probably due to larger surface area of the adsorbent being available at the beginning for the adsorption of Cr(III) (Sumathi et al. 2004).

Measurement of percentage Cr(III) adsorption as a function of time indicates that percentage adsorption of Cr(III) increased with an increase in contact time and attained equilibrium after 15 min irrespective of the concentration of Cr(III). But, the time taken to reach apparent equilibrium was increased at higher initial concentrations.

It should be noted that the removal trend is almost identical irrespective of the initial Cr(III) dose. This is presumably due to higher dose of adsorbent used in this experiment (adsorbent dose 40 g/L) and may be also due to smaller differences in the initial Cr(III) dose (40, 50, 60 mg/L).

### Effect of initial pH

**Fig. 2:**
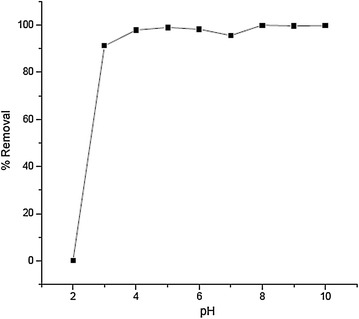
Percent removal of Cr(III) as a function of initial pH (adsorbent dose 20 g/L, initial Cr(III) concentration 40 mg/L, contact time 60 min)

The pH value of the solution is an important factor that controls the uptake of Cr(III). The experimental results revealed that the percentage adsorption of Cr(III) increased as the pH increases and reached 98 % at pH 4 (Fig. 2). When pH was decreased below 3, the percent removal decreased, with only 0.1 % removal at pH 2 indicating that the adsorbent is totally ineffective at very low pH.

Adsorption at different pH can be explained by the species distribution of chromium in water and the nature of adsorbent surface. In acidic pH, the predominant species of Cr(III) cations are: Cr^3+^, CrOH^2+^ and CrOH_2_^+^ and under acidic conditions, the surface of the adsorbent becomes protonated and hence there is a decrease in the electrostatic attraction between the Cr(III) species and the adsorbent surface, with a consequent decrease in percentage adsorption. But as pH increases, the adsorbent surface becomes less protonated and will have strong attraction for cationic species of Cr(III).

Another possibility for the increase in percentage removal of Cr(III) with increasing pH is formation of insoluble hydroxide precipitate at higher pH values. Generally adsorption of Cr(III) was higher at higher pH and decreased with decreasing pH for the reasons explained above. The removal efficiency curve (Fig. 2) shows that aluminum oxide hydroxide is effective over a wide pH range (3–10).

In the present study, the equilibrium pH was found to be greater than that of initial pH for initial pH values <6. But, the equilibrium pH values were decreased to around 6 for initial pH values >6. The resultant equilibrium pH as a function of initial pH is shown in Table 1. Increase in pH can be explained by protonation of adsorbent. When the initial pH values are <6, the adsorbent acts as a base and gets protonated. This process decreased H^+^ ion concentration and resulted in increased pH. But, the adsorbent acts as an acid and gets deprotonated when the initial pH values are >6. As a result, pH was decreased. Decrease in the pH may also be due to formation of insoluble Cr hydroxide which takes away OH^−^ ion from the solution.

**Table 1:** Equilibrium pH as a function of initial pH

Initial pH	Equilibrium pH
2	4.60
3	5.28
4	5.57
5	5.68
6	5.80
7	6.30
8	6.00
9	6.10
10	6.13

### Effect of adsorbent dose

**Fig. 3:**
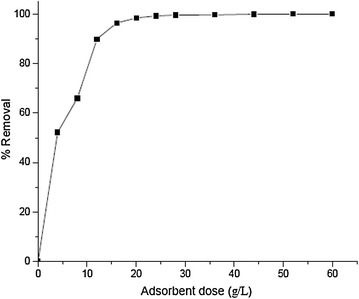
Percent removal of Cr(III) as a function of amount of adsorbent [initial Cr(III) concentration 40 mg/L, contact time 60 min, initial pH 3.8]

The percentage adsorption of Cr(III) was studied by increasing the adsorbent dose from 4 to 60 g/L at 40 mg/L initial concentration of Cr(III) solution. The results indicated more than 98 % removal of Cr(III) with 20 g/L adsorbent dose after 60 min of contact time. It is observed that within the range of adsorbent dose studied, the percent removal increases with an increase in the amount of adsorbent up to an optimum amount of the adsorbent beyond which the percent removal remains nearly the same (Fig. 3). The increase in the percentage adsorption with increase in the adsorbent dosage is due to the increase in the number of adsorption sites.

### Adsorption isotherm

**Fig. 4:**
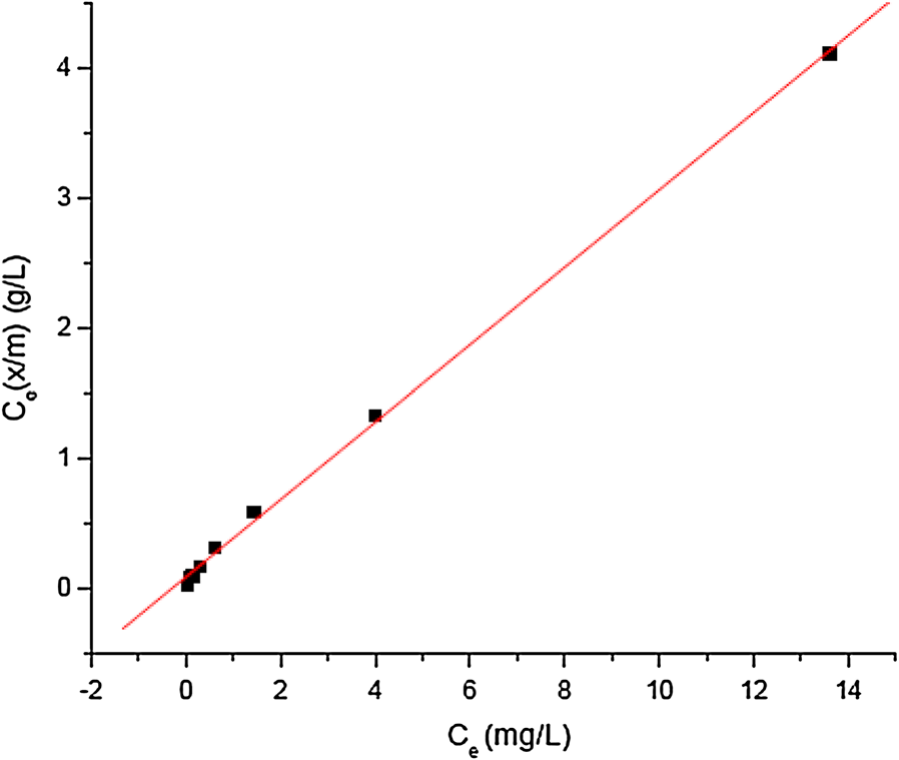
Langmuir adsorption isotherm for adsorption of Cr(III) on the aluminum oxide hydroxide [initial Cr(III) concentration 40 mg/L, contact time 60 min, initial pH 3.8]

The adsorption equilibrium data obtained at a fixed initial Cr(III) concentration and varying adsorbent dose have been fitted into the linear form of Langmuir and Freundlich adsorption isotherms. The simplest adsorption isotherm, Langmuir isotherm, is based on the assumptions that every adsorption site is equivalent and that the ability of a particle to bind there is independent of whether or not adjacent sites are occupied. Linear form of Langmuir adsorption equation is given by:

$${\text{x/m}} = 1 / {\text{x}}_{\text{m}} + 1 / {\text{b}}\;{\text{x}}_{\text{m}} {\text{C}}_{\text{e}} \;{\text{or}}\;{\text{C}}_{\text{e}} \left( {\text{x/m}} \right) = {\text{C}}_{\text{e}} / {\text{x}}_{\text{m}} + 1 / {\text{bx}}_{\text{m}}$$

which is in the form of y = bx + a with slope 1/x_m_ and y-intercept 1/bx_m_. Where x/m is adsorption capacity, amount of solute adsorbed (in mg) per amount of adsorbent (in g), x_m_ is amount of solute adsorbed per unit weight of adsorbent required for monolayer coverage of the surface also called monolayer capacity and b is a constant related to the heat of adsorption and C_e_ is equilibrium concentration (Sarin and Pant 2006). x_m_ and b were determined from the slope and intercept of the plot to be 3.36 mg/g and 3.35 L/mg, respectively. The adsorption capacity obtained in this experiment is in agreement with the results reported in the literature (maximum sorption capacity in the range of 3.23–11.8 mg/g (Selvaraj et al. 2003). Linear plot of C_e_/(x/m) versus C_e_ (Fig. 4) with 0.9994 regression coefficient (R^2^) showed that the adsorption equilibrium obeyed Langmuir model exhibiting monolayer adsorption.

**Fig. 5:**
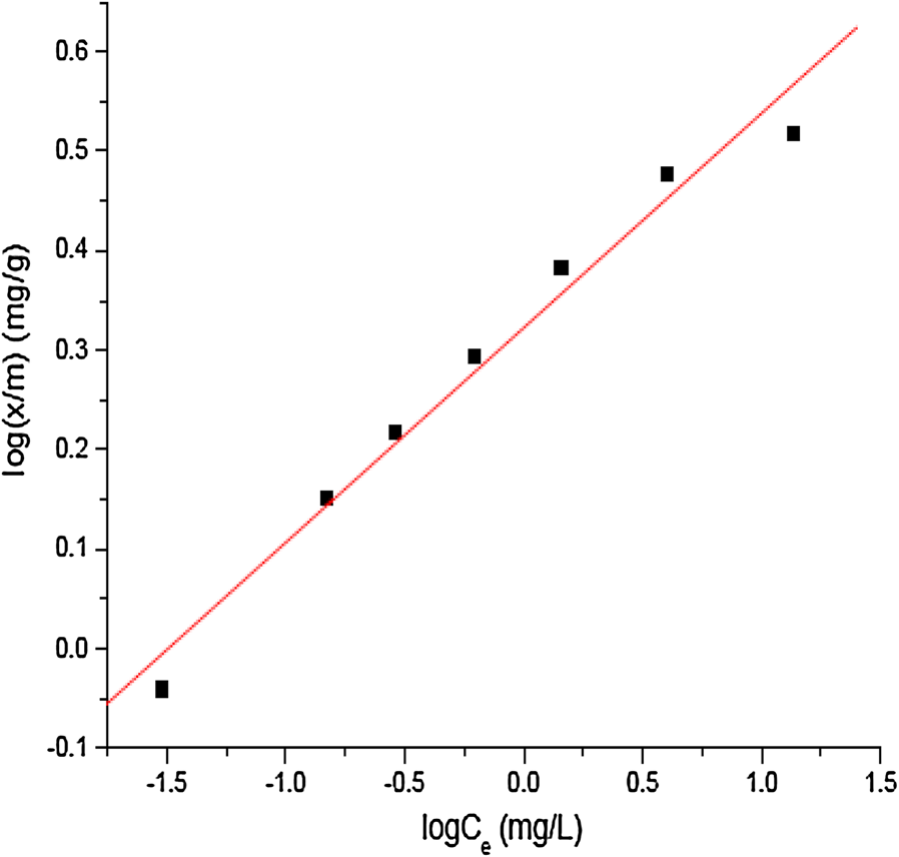
Freundlich adsorption isotherm for adsorption of Cr(III) on to aluminum oxide hydroxide [initial Cr(III) concentration 40 mg/L, contact time 60 min, initial pH 3.8]

Freundlich adsorption isotherm, which assumes heterogeneous surface conditions, states that the adsorption capacity, x/m, is a function of the equilibrium concentration of the solute. The linear form of Freundlich equation is expressed as:

$${ \log }\left( {\text{x/m}} \right) = { \log }\left( {{\text{K}}_{\text{f}} } \right) + 1 / {\text{n}}\;{ \log }\left( {{\text{C}}_{\text{e}} } \right)$$

where K_f_ is constant related to adsorption capacity, 1/n is constant related to adsorption intensity (Salimi et al. 2006). Linear plot of log(x/m) versus log(C_e_) (Fig. 5) with 0.988 regression coefficient showed that the adsorption equilibrium also obeyed Freundlich model exhibiting heterogeneous surface conditions. K_f_ and 1/*n* values as obtained from the plot (Fig. 5) are 2.09 mg/g and 0.22 L/g, respectively. The low value of 1/*n* (<1), indicates a greater adsorption efficiency of the newly developed adsorbent. These values are comparable with several published literature reported for various adsorbents (Chakir et al. 2002; Sarin and Pant 2006).

### Effect of initial concentration

**Fig. 6:**
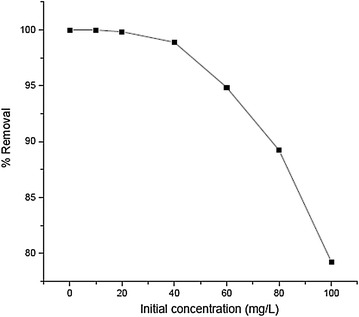
Percent removal of Cr(III) as a function of initial concentration (contact time 60 min, adsorbent dose 20 g/L, initial pH 3.8)

Adsorption experiments at varying initial Cr(III) concentrations from 10 to 100 mg/L were performed with fixed doses (20 g/L) of adsorbent. The results indicates that percentage Cr(III) removal decreases as the initial concentration of Cr(III) was increased. Cr(III) removal ranged from 100 to 79 % at initial Cr(III) concentration of 10–100 mg/L (Fig. 6). This can be explained by the fact that all the adsorbents had a limited number of active sites, which would have become saturated above a certain concentration. Since percent removal = (C_o_ − C_e_)/C_o_, another reason for decrease in percent removal is larger increase in the denominator (C_o_) value in comparison to that of (C_o_ − C_e_) value. But, the actual amount of chromium removed (mg) per gram of the adsorbent is lager for higher concentration (Csobán and Joó 1999; Yu et al. 2003).

In general, the optimum conditions for the removal of Cr(III) were found to be at pH 4–6 with 40 g/L adsorbent dose at 60 min of contact time.

### Application of the method to real sample

Efficiency of the adsorbent, aluminum oxide hydroxide, for the removal of chromium was tested using Hafde tannery waste effluent (Addis Ababa, Ethiopia). The chromium concentration in the effluent at the discharge point was very high (5520 mg/L). Adsorption experiments on this real sample were carried out at two different pH values (Tables 2, 3) at pH 3.8, which is the pH of the waste effluent and at pH 5. And the adsorbent was found almost equally effective at the two pH values. Thus, the adsorbent can be directly applied without any pH adjustment. As the waste effluent is highly concentrated adsorption experiments were carried out after dilution (25 times). Even after diluting the effluent, it is still very concentrated and contains 221 mg/L of chromium. That is why percent adsorptions (Tables 2, 3) seem small (74 and 76 %). But, the actual amount removed per gram of the adsorbent is as high as that of standard aqueous solution prepared in the laboratory.

**Table 2:** Percent removal of chromium from the real sample solution (diluted 25 times) (contact time 60 min, adsorbent dose 40 g/L, initial pH 3.8)

Exp. no.	Initial conc. (mg/L)	Final conc. (mg/L)	Average final conc. (mg/L)	% Removal
1	221	56.2		
2	221	56.8	56.6 ± 0.3	74.4
3	221	56.8

**Table 3:** Percent chromium removal from the real sample solution (diluted 25 times) (contact time 60 min, adsorbent dose 40 g/L, initial pH 5)

Exp. no.	Initial conc. (mg/L)	Final conc. (mg/L)	Average final conc. (mg/L)	% Removal
1	221	51.9		
2	221	53.6	52.8 ± 0.8	76.1
3	221	53.0		

## Conclusion

In this study, an efficient adsorption method was developed for the removal of trivalent chromium from the waste water based on batch mode of adsorption experiments. Aluminum oxide hydroxide was found to be very effective adsorbent for removing trivalent chromium from waste water. Effects of different parameters (such as contact time, initial pH, amount of adsorbents, and initial Cr(III) concentration) were studied for the maximum adsorption of Cr(III) on aluminum oxide hydroxide. The study revealed more than 99 % removal of Cr(III) with 1 g of adsorbent in 50 mL of 40 mg/L Cr(III) solution over a wide range of initial pH (3–10). The adsorption equilibrium data obtained at a fixed initial concentration and varying adsorbent dose were well fitted into the Langmuir and Freundlich adsorption isotherms. The proposed adsorbent, aluminum oxide hydroxide, was applied to the removal of Cr(III) from tannery waste effluent (Addis Ababa, Ethiopia) and found to be very efficient. Thus the adsorbent can be applied to the removal of chromium from waste effluent.
